# Characteristics and Potential Challenges of Digital-Based Interventions for Children and Young People: Scoping Review

**DOI:** 10.2196/45465

**Published:** 2023-04-14

**Authors:** Jinsoo Yun, Jaeyong Shin, Hyerim Lee, Dai-Jin Kim, In-Young Choi, Meelim Kim

**Affiliations:** 1 College of Nursing Yonsei University Seoul Republic of Korea; 2 Department of Preventive Medicine College of Medicine Yonsei University Seoul Republic of Korea; 3 Department of Psychology College of Liberal Arts Yonsei University Seoul Republic of Korea; 4 Department of Psychiatry College of Medicine The Catholic University of Korea Seoul Republic of Korea; 5 Department of Medical Informatics College of Medicine The Catholic University of Korea Seoul Republic of Korea; 6 Herbert Wertheim School of Public Health and Human Longevity Science University of California San Diego San Diego, CA United States; 7 Health IT Center Yonsei University Health System Seoul Republic of Korea

**Keywords:** digital health, digital intervention, children and young people, ethical challenge, interpersonal challenge, societal challenge

## Abstract

**Background:**

Digital health technologies are becoming increasingly available to children and young people and their families. However, there are no scoping reviews that provide both an overview of the characteristics of digital interventions for children and young people and potential challenges to be considered when developing and implementing them.

**Objective:**

This study aimed to systematically review scientific publications to identify the current characteristics and potential complications of digital interventions for children and young people.

**Methods:**

This scoping review was conducted using the framework of Arksey and O’Malley and adheres to the PRISMA (Preferred Reporting Items for Systematic Reviews and Meta-Analyses) guidelines for scoping reviews. A search of 5 databases (PubMed, Scopus, Embase, MEDLINE, and CINAHL) and Google Scholar was performed for eligible clinical trials published between January 1, 2018, and August 19, 2022.

**Results:**

The initial search of the 5 databases yielded 3775 citations; duplicates and those not meeting the inclusion criteria were eliminated. In total, 34 articles were included in the final review and relevant information, such as the descriptive characteristics and potential challenges, were classified. Mental health (26/34, 76%) was the most common target for the digital intervention for children and young people, exceeding physical health (8/34, 24%) by more than 3 times. In addition, a substantial number of digital interventions were dedicated solely to children and young people. Digital interventions for children and young people were more likely to be delivered via computers (17/34, 50%) rather than smartphones (13/34, 38%). More than one-third of the studies (13/34, 38%) applied cognitive behavioral theory as the theory of digital interventions. The duration of the digital intervention for children and young people was more likely to vary depending on the target user rather than the target disease. Intervention components were classified into 5 categories: guidance, task and activity, reminder and monitoring, supportive feedback, and reward system. Potential challenges were subcategorized into ethical, interpersonal, and societal challenges. For ethical challenges, the consent of children and young people or caregivers, potential risk of adverse events, and data privacy issues were considered. For interpersonal challenges, the engagement of children and young people was affected by the preference or barrier of caregivers to participate in studies. For societal challenges, restricted ethnicity in recruitment, limited availability of digital technology, differences in internet use patterns between girls and boys, unified clinical settings, and language barriers were described.

**Conclusions:**

We identified potential challenges and provided suggestions about ethical, interpersonal, and societal aspects to consider when developing and deploying digital-based interventions for children and young people. Our findings provide a thorough overview of the published literature and may serve as a comprehensive, informative foundation for the development and implementation of digital-based interventions for children and young people.

## Introduction

### Background

Digital therapeutics (DTx) uses digital technologies to deliver high-fidelity evidence-based treatments at scale to improve health outcomes and reduce the burden on health care professionals [1]. One of the primary benefits of DTx is its ability to overcome geographical and time-related barriers to health care accessibility. Children and young people have not been left behind in the advancement of digital technology in health care. As smartphones are becoming more available to children and families, DTx may improve and maintain the health of children and young people.

Providing health care for children and young people, a socially vulnerable population, requires a specific skill set owing to their distinct characteristics and needs [2]. In children and young people, the communication skills necessary for making medical decisions and understanding the information related to medical treatment may vary depending on their verbal language development level [3]. In addition, children and young people’s emotional (eg, anxiety or depression) and physical (eg, pain or a range of medications) conditions may affect their level of commitment to the treatment [3]. Finally, the children and young people’s cultural and religious background may influence how much they value medical treatment and how they interact with health care professionals [3]. Therefore, when conducting clinical practice or research on children and young people, various factors must be carefully considered.

Children and young people are also known for being early adopters of digital technologies, including digital health technologies. Hence, the advancement of digital technologies dedicated to children and young people offers considerable benefits and is frequently discussed in the literature. However, as children and young people are susceptible to manipulation, especially via digital devices and tools, digital platforms have terms of service that are specifically adapted to children and young people [4-6]. For example, the Google Play Store necessitates an in-app reminder within all social media apps to stay safe on the web and be aware of the real-world risks of web-based activities [4]. The Apple App Store requires parental permission to protect children’s privacy and prevent them from engaging in commerce or following external links to websites, social networks, or other apps without their parent or guardian’s knowledge [5,6]. Each of the Google Play Store and Apple App Store app review guidelines provides specific details regarding their requirements. Therefore, when developing digital interventions for children and young people, a holistic and careful approach based on the distinct guidelines of the digital platform is necessary.

Although there is growing interest in digital interventions for children and young people, we have not found reviews that provide an overview of the characteristics of digital interventions for children and young people, as well as the potential challenges to be considered when developing them. Thus, we performed a scoping review that summarizes the current characteristics of the digital interventions dedicated to children and young people, such as their medical specialties, target users, and features, and the potential challenges associated with their development, as described in peer-reviewed literature.

### Objective

Our study aimed to provide a broader perspective that summarizes the characteristics of digital interventions and potential challenges for children and young people, as published in the peer-reviewed literature. The research questions were as follows:

1. What are the characteristics of digital interventions dedicated to children and young people?
2. What potential challenges should be considered when implementing digital interventions for children and young people?

## Methods

### Design

This scoping review used the framework of Arksey and O’Malley [7]. This scoping review is reported based on the PRISMA-ScR (Preferred Reporting Items for Systematic Reviews and Meta-Analyses extension for Scoping Reviews) guidelines [8]. The completed PRISMA-ScR checklist is presented in Multimedia Appendix 1.

### Identifying Relevant Studies

We performed a search in 5 databases (PubMed, Scopus, Embase, MEDLINE, and CINAHL) and Google Scholar to identify studies that examined at least 1 digital intervention targeting children and young people with specific medical problems or their caregivers. The analyses were limited to clinical trials published in English between January 1, 2018, and August 19, 2022. The complete search strategy is outlined in Multimedia Appendix 2.

### Study Selection

The inclusion and exclusion criteria were applied to screen titles, abstracts, and full-text papers for study selection (Multimedia Appendix 3). When there was a disagreement over study selection, consensus was reached through discussion.

### Charting the Data

The selected studies were analyzed to identify the characteristics and possible complications of digital interventions for children and young people. On the basis of data items, relevant information was extracted and charted into a specific form. The form included the following data items: authors, year of publication, title, aim, population, target disease, user, study design, duration of intervention, intervention protocol, and results.

### Collating, Summarizing, and Reporting the Results

To address the research questions, charted data were used as supplementary sources and full-text papers were read multiple times. Results were independently collected from each article, and cross-validation was performed. Two categories, descriptive characteristics and potential challenges, were used to group the research results. Reporting of the results about the identified characteristics and potential challenges of digital intervention for children and young people was done using both narrative and numerical descriptions.

## Results

### Overview

**Figure 1:**
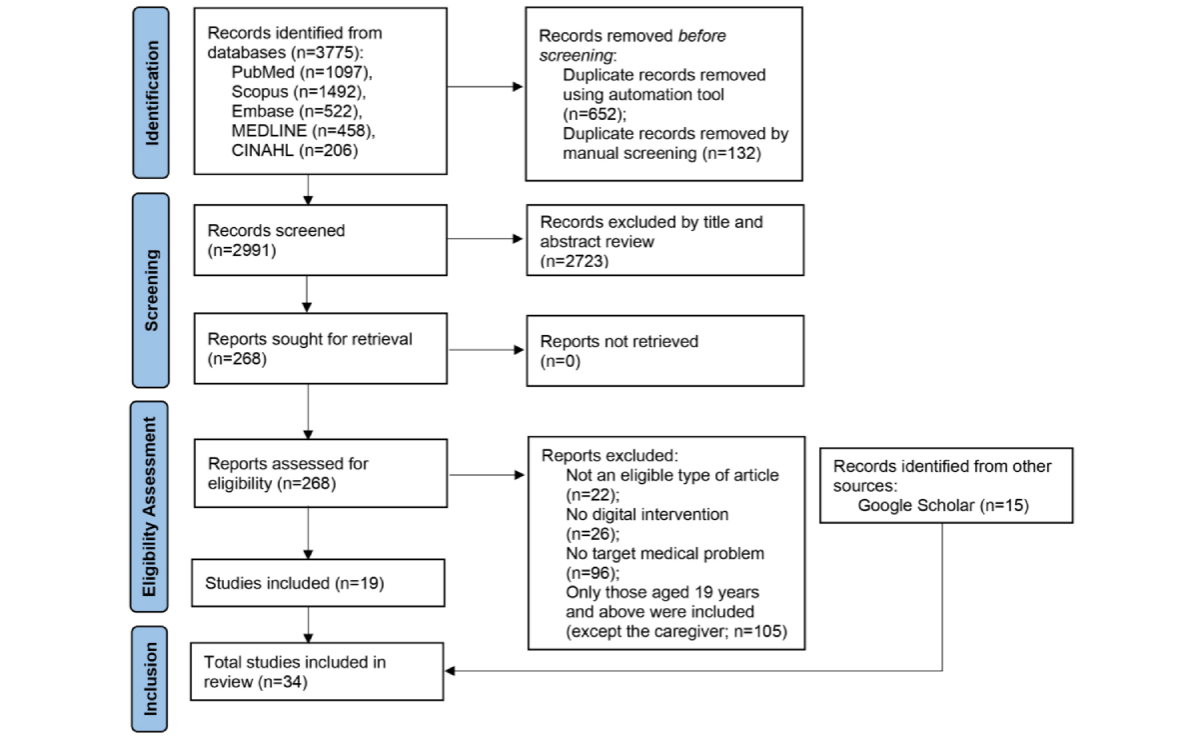
PRISMA (Preferred Reporting Items for Systematic Reviews and Meta-Analyses) flowchart.

The following 4 review phases were conducted based on the PRISMA (Preferred Reporting Items for Systematic Reviews and Meta-Analyses) flowchart (Figure 1): identification, screening, eligibility assessment, and final synthesis. Our search query initially retrieved 3775 articles. A total of 652 articles were identified during the automatic duplicate removal process, and 132 articles were manually identified as duplicates and removed. Of the remaining 2991 articles, 2723 were removed based on title and abstract screening and the inclusion and exclusion criteria (Multimedia Appendix 3). The remaining 268 articles were assessed for eligibility using full-text screening, and 19 articles were eligible; 15 articles identified on Google Scholar were included in this review. Finally, 34 articles were included in the analysis.

### Descriptive Characteristics

The percentage of digital interventions targeting mental disorders was higher than that targeting physical diseases (26/34, 76% vs 8/34, 24%; Table 1). The Diagnostic and Statistical Manual of Mental Disorders, Fifth Edition criteria were used to differentiate between mental and physical disorders. If the Diagnostic and Statistical Manual of Mental Disorders, Fifth Edition criteria were satisfied, it was classified as a mental disorder. Regarding the target users, 85% (29/34) of interventions targeted children and young people, 6% (2/34) targeted their caregivers, and 9% (3/34) targeted both groups.

Most of the digital interventions were web based (17/34, 50%) or mobile app based (13/34, 38%). A total of 4 studies combined 2 different modalities to deliver the intervention (eg, web-based + eye tracker [9] and mobile app–based + Google Glass; Alphabet Inc [10]). The details are listed in Table 2.

More than one-third of the studies (13/34, 38%) applied cognitive behavioral theory as the theory of digital interventions. A total of 5 studies were based on behavior change theory, and 4 studies were based on cognitive theory. Three studies were based on applied behavior analysis. Guideline-based intervention was used in 3 studies. One study applied 3 different theories, namely social learning theory, family systems theory, and cognitive behavioral theory, to inform their intervention [11]. The psychodynamic theory was used in 1 study [12]. The details are listed in Table 3.

Regarding the active intervention duration, which refers to users actively receiving the intervention components, digital interventions ranged from a single session to 28 weeks (average 8.8, SD 6.49 weeks) for mental disorders and from 4 to 24 weeks (average 11, SD 6.05 weeks) for physical disorders. Digital interventions ranged from a single session to 24 weeks (average 8.1, SD 5.09 weeks) for children and young people, 11 to 28 weeks (average 19.5, SD 12.02 weeks) for caregivers, and 8 to 24 weeks (average 14, SD 8.72 weeks) when targeting both children and young people and their caregivers. The details are listed in Table 4.

**Figure 2:**
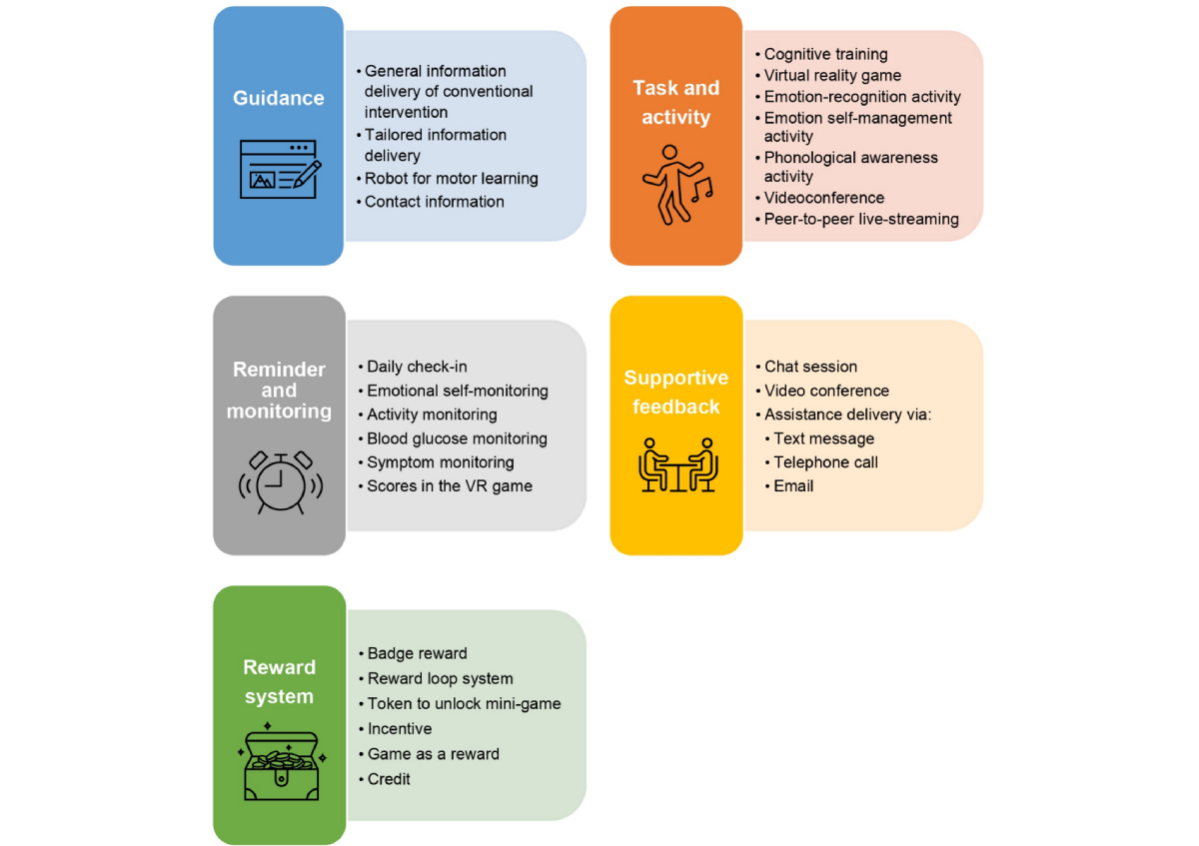
Examples and proportions of each intervention component in digital interventions for children and young people.

By adapting existing frameworks to the design of digital interventions [13], the intervention components were classified into the following 5 categories: guidance, task and activity, reminder and monitoring (RM), supportive feedback (SF), and reward system (RS; Figure 2).

Guidance components provided users with educational information and were included in 22 studies. Of these, 19 provided users with education, for example, cognitive behavioral therapy, behavioral interventions, social story interventions, parent training programs, coping skills, or physical activity education [11,12,14-30]. Two studies provided users with tailored educational information, including personalized health and wellness information for someone with Down syndrome [31] and nutritional information with individualized meal plans [32]. One study used a robot to guide children in engaging in the repetitive motions required for motor learning [33]. One study provided users with contact information for mental health support services [25].

Task and activity components involved engagement or participation within the intervention [13] and were included in 17 studies. Four offered cognitive training [34-37], 1 offered a virtual reality game [33], and 2 offered emotion-recognition activities [9,10]. Seven studies provided users with homework exercises to help them practice learning [11,12,14,18,21,26,38]. To enhance emotional regulation skills, some interventions used several activities for self-management of overwhelming emotions [39] or reading and writing tasks for depression, anxiety, and well-being [40]. Furthermore, other studies included phonological awareness activities [38], videoconferencing with a therapist [30], peer-to-peer live streaming [15], or dance sessions [27].

In the RM component, users’ progress or status was monitored. Reminders were sent via SMS text messages [15,35], emails [11,31,34,41], or a digital intervention platform [11,12,14,34,37]. In total, 13 studies used self-monitoring, including daily check-ins [11], emotional self-monitoring [25], activity and blood glucose monitoring [27], symptom monitoring [29], bolus and nutrient content calculating [42], web-based assessment [43], assessment of depressive symptoms and sleep diary [20], daily mood queries [22], mood history [25], mood activity diary [18], ketosis monitoring [32], current health data recording [32], and scores in the virtual reality game [33]. The other 6 studies relied on monitoring features to ensure user compliance [9,15,16,21,34,37]. A list of the overall summary of the intervention components used in each study on digital interventions for children and young people or caregivers is provided in Multimedia Appendix 4 [9-12,14-43].

SF features enabled users to receive personalized feedback on their progress. One study provided SF to users via SMS text messages [43], 5 via telephone calls [19-21,26,35], 3 via emails [14,26,42], 3 via a digital intervention platform [12,29,31], and 1 via a human coach [33]. Some studies included chat sessions [18,23] and videoconferences with trained therapists [30]. Three studies offered >1 method of assistance delivery [12,24,27].

RS components are mechanisms that provide reinforcements to enhance the user’s behavioral change. Different types of rewards were used in 6 studies, including badge and RSs [11], reward loops [34], tokens to unlock minigames [9], incentives [35], unlocking the game Balloon Blast as a reward [39], and credits [43].

**Table 1:** Number and proportion of target diseases and target users (N=34).

		Studies, n (%)	References
**Target disease**			
	Mental disorder	26 (76)	Moor et al [14], Kollins et al [34], Sosnowski et al [9], Zheng et al [15], Hanrahan et al [16], Chung et al [31], Ko et al [36], Edridge et al [39], Dobias et al [17], Haug et al [43], Topooco et al [18,23], Jesus et al [38], Aspvall et al [19], Cliffe et al [20], Voss et al [10], Sourander et al [21], Ranney et al [22], Gallen et al [37], Khan et al [24], Kenny et al [25], Lenhard et al [26], Osborn et al [40], Nordh et al [28], Lindqvist et al [12], and Wade et al [30]
	Physical disease	8 (24)	Palermo et al [11], Hardy et al [35], Klee et al [42], Alfonsi et al [41], Lei et al [32], Shedrief et al [33], Knox et al [27], and Schmidt et al [29]
**Target user**			
	Children or adolescents only	29 (85)	Kollins et al [34], Sosnowski et al [9], Zheng et al [15], Hanrahan et al [16], Hardy et al [35], Ko et al [36], Klee et al [42], Edridge et al [39], Dobias et al [17], Haug et al [43], Alfonsi et al [41], Topooco et al [18,23], Jesus et al [38], Aspvall et al [19], Cliffe et al [20], Voss et al [10], Lei et al [32], Shedrief et al [33], Ranney et al [22], Gallen et al [37], Khan et al [24], Kenny et al [25], Lenhard et al [26], Osborn et al [40], Knox et al [27], Nordh et al [28], Schmidt et al [29], and Lindqvist et al [12]
	Caregivers only	2 (6)	Chung et al [31] and Sourander et al [21]

**Table 2:** Number and proportion of intervention modalities (N=34).

Intervention modality		Studies, n (%)	References
**App (mobile) vs web (computer)**			
App (mobile)		13 (38)	Palermo et al [11], Kollins et al [34], Hanrahan et al [16], Ko et al [36], Klee et al [42], Edridge et al [39], Haug et al [43], Alfonsi et al [41], Jesus et al [38], Lei et al [32], allen et al [37], Kenny et al [25], and Schmidt et al [29]
Web (computer)		17 (50)	Moor et al [14], Zheng et al [15], Chung et al [31], Hardy et al [35], Dobias et al [17], Topooco et al [18,23], Aspvall et al [19], Cliffe et al [20], Sourander et al [21], Ranney et al [21], Khan et al [24], Lenhard et al [26], Osborn et al [40], Nordh et al [4], Lindqvist et al [12], and Wade et al [30]
Combined modalities		4 (12)	Sosnowski et al [9], Voss et al [10], Knox et al [27], and Shedrief et al [33]

**Table 3:** Number and proportion of intervention backgrounds (N=34).

Intervention background	Studies, n (%)	References
Cognitive behavioral theory	13 (38)	Moor et al [14], Palermo et al [11], Edridge et al [39], Dobias et al [17], Topooco et al [18,23], Aspvall et al [19], Cliffe et al [20], Ranney et al [21], Lenhard et al [26], Nordh et al [4], Schmidt et al [29], and Wade et al [30]
Behavior change theory	5 (15)	Sourander et al [21], Khan et al [24], Kenny et al [25], Knox et al [27], and Haug et al [43]
Cognitive theory	4 (12)	Kollins et al [34], Hardy et al [35], Ko et al [36], and Gallen et al [37]
Applied behavior analysis	3 (9)	Sosnowski et al [9], Hanrahan et al [16], and Voss et al [10]
Guideline-based intervention	3 (9)	Chung et al [31], Jesus et al [38], and Shedrief et al [33]
Social learning theory	1 (3)	Palermo et al [11]
Family systems theory	1 (3)	Palermo et al [11]
Psychodynamic theory	1 (3)	Lindqvist et al [12]
Motivational interviewing	1 (3)	Ranney et al [22]
Mindfulness	1 (3)	Edridge et al [39]
Not specifically defined	5 (15)	Zheng et al [15], Alfonsi et al [41], Lei et al [32], Osborn et al [40], and Klee et al [42]

**Table 4:** Numeric description of range and mean duration of active intervention.

Active intervention duration		Values, range	Duration (weeks), mean (SD)	References
**Target disease**				
	Mental disorder	Single session-28 weeks	8.8 (6.49)	Moor et al [14], Kollins et al [34], Sosnowski et al [9], Zheng et al [15], Hanrahan et al [16], Chung et al [31], Ko et al [36], Edridge et al [39], Dobias et al [17], Haug et al [43], Topooco et al [18,23], Jesus et al [38], Aspvall et al [19], Cliffe et al [20], Voss et al [10], Sourander et al [21], Ranney et al [22], Gallen et al [37], Khan et al [24], Kenny et al [25], Lenhard et al [26], Osborn et al [40], Nordh et al [28], Lindqvist et al [12], and Wade et al [30]
	Physical disease	4 weeks-24 weeks	11 (6.05)	Palermo et al [11], Hardy et al [35], Klee et al [42], Alfonsi et al [41], Lei et al [32], Shedrief et al [33], Knox et al [27], and Schmidt et al [29]
**Target user**				
	Children or adolescents only	Single session-24 weeks	8.1 (5.09)	Kollins et al [34], Sosnowski et al [9], Zheng et al [15], Hanrahan et al [16], Hardy et al [35], Ko et al [36], Klee et al [42], Edridge et al [39], Dobias et al [17], Haug et al [43], Alfonsi et al [41], Topooco et al [18,23], Jesus et al [38], Aspvall et al [19], Cliffe et al [20], Voss et al [10], Lei et al [32], Shedrief et al [33], Ranney et al [22], Gallen et al [37], Khan et al [24], Kenny et al [25], Lenhard et al [26], Osborn et al [40], Knox et al [27], Nordh et al [28], Schmidt et al [29], and Lindqvist et al [12]
	Caregivers only	11 weeks-28 weeks	19.5 (12.02)	Chung et al [31] and Sourander et al [21]
	Children or adolescents and caregivers	8 weeks-24 weeks	14 (8.72)	Moor et al [14], Palermo et al [11], and Wade et al [30]

### Potential Challenges

#### Ethical Challenges

Ethical challenges were categorized into two themes: (1) consent and (2) potential risk (Table 5). Children and young people or caregivers must consent to proceed with clinical trials or digital interventions. In addition, potential risks include adverse outcomes and secondary unintended harmful effects of the intervention such as data rights and privacy issues.

**Table 5:** Potential challenges of digital interventions for children and young people or caregivers.

Type of challenge and content						References
**Ethical challenges**						
	**Consent**					
		From the caregiver and children and young people				Palermo et al [11], Kollins et al [34], Sosnowski et al [9], Hanrahan et al [16], Chung et al [31], Hardy et al [35], Klee et al [42], Haug et al [43], Alfonsi et al [41], Topooco et al [18], Aspvall et al [19], Cliffe et al [20], Voss et al [10] Ranney et al [22], Gallen et al [37], Khan et al [24], Kenny et al [25], Lenhard et al [26], Osborn et al [40], Knox et al [27], Nordh et al [28], Schmidt et al [29], and Wade et al [30]
		From caregiver only				Zheng et al [15], Ko et al [36], Jesus et al [38], Lei et al [32], Sourander et al [21], and Shedrief et al [33]
		From children and young people only				Dobias et al [17], Topooco et al [23], and Lindqvist et al [12]
	**Potential risk**					
		**Adverse event**				
			**Psychological**			
				Anxiety, depression, loneliness, anger, distress, shame, suicide attempt, frustration, emotional reaction, and aggression	Moor et al [14], Topooco et al [18,23], Lindqvist et al [12], Nordh et al [28], and Kollins et al [34]	
			**Physical**			
				Headache, dizziness, nausea, and *adverse reaction to a digital tool*	Kollins et al [34] and Voss et al [10]	
			**Not specifically defined**			
				Few side effects	Hardy et al [35]	
		**Data privacy**				
			Deidentified data sharing publicly available		Dobias et al [17]	
			Deidentified data sharing available upon specific request		Kollins et al [34], Voss et al [10], Gallen et al [37], Knox et al [27], and Nordh et al [28]	
			Data are not available in any case		Lenhard et al [26]	
			Not specifically defined		Moor et al [14], Palermo et al [11], Sosnowski et al [9], Zheng et al [15], Hanrahan et al [16], Chung et al [31], Hardy et al [35], Ko et al [36], Klee et al [42], Edridge et al [39], Haug et al [43], Alfonsi et al [41], Topooco et al [18,23], Jesus et al [38], Aspvall et al [19], Cliffe et al [20], Lei et al [32], Sourander et al [21], Shedrief et al [33], Ranney et al [22], Khan et al [24], Kenny et al [25], Osborn et al [40], Schmidt et al [29], Lindqvist et al [12], and Wade et al [30]	
**Interpersonal challenges**						
	**Caregiver attitude**					
		Biased recruitment toward reliable, frequent attendees			Khan et al [24]	
	**Caregiver involvement**					
		Caregiver involvement for children and young people access to digital resources			Wade et al [30]	
		Caregiver involvement in intervention of family history–related target disease			Lei et al [32]	
	**Caregiver acceptability**					
		Time commitment to the intervention or accessibility to clinics for assessments			Hardy et al [35]	
**Societal challenges**						
	**Ethnicity**					
		Restricted ethnicity in recruitment			Palermo et al [11], Chung et al [31], Dobias et al [17], and Knox et al [27]	
	**Technology availability**					
		Limited availability of digital technology in rural communities or situations			Sosnowski et al [9], Zheng et al [15], Dobias et al [17], Osborn et al [40], and Schmidt et al [29]	
	**Sex**					
		Differences in internet use patterns and preferences between girls and boys			Topooco et al [18,23]	
	**Clinical setting**					
		Specific clinical settings to be considered in generalizing the outcome			Lenhard et al [26] and Aspvall et al [19]	
	**Language**					
		Non-English speakers were excluded from research			Ranney et al [22]	

#### Consent

Both caregivers and children and young people consented to participate in the clinical trials in 23 studies [9-11,16,18-20,22,24-31,34,35,37,40-43]. In 6 studies [15,21,32,33,36,38], only caregivers provided consent; 3 of these studies [33,36,38] included only children and young people aged <7 years. In 3 studies [12,17,23], only children and young people provided consent. Two reasons for waiving caregiver consent were provided. Individuals aged 15 to 17 years can consent to research without parental involvement in Sweden, assuming they are mature enough to freely participate in studies while being aware of the potential adverse consequences [23]. In addition, parental consent was waived to reduce logistical barriers and increase willingness to participate in the research among marginalized adolescents (lesbian, gay, bisexual, transgender, and queer youth), who might otherwise be underrepresented in mental health treatment research [17]. The other 2 studies (1 conducted in a school setting [39] and the other in a primary care setting [14]) did not provide details regarding obtaining consent.

#### Potential Risks

Although most participants showed improved outcomes, interventions in 3 studies had unexpected adverse effects on some participants, particularly those with psychological issues (ie, increased levels of anxiety [14] or depression [18,23]). In addition, various psychological concerns (eg, loneliness, anger, distress, and shame) were reported by participants with no serious adverse events in 1 study [12]. A suicide attempt was found in 1 web-based intervention targeting children and young people with social anxiety disorder [28]. Both psychological (eg, frustration, emotional reaction, and aggression) and physical (eg, headache, dizziness, and nausea) concerns have been reported in the app-based intervention for attention-deficit/hyperactivity disorder [34]. Furthermore, an adverse reaction was reported in the intervention using wearable glass devices for children with autism spectrum disorder [10]. Another study reported few side effects using the Side Effects Rating Scale [35].

To protect participant data, databases were on a secure platform [9,11,14,17,22] or otherwise data were anonymized before statistical evaluation [15,34]. Regarding data availability, deidentified data from 1 study were publicly available [17], whereas deidentified data from other studies were available upon approval of the proposed use of the data [10,27,28,34,37]. Conversely, data from 1 study were unavailable under any circumstance owing to European data regulation restrictions by the General Data Protection Regulation [26]. In 1 study, a participant dropped out of the digital intervention because they felt uncomfortable sharing the requested information [31].

#### Interpersonal Challenges

Interpersonal factors, such as family attitudes and decision-making regarding digital intervention, can impact children and young people participation or clinical outcomes (Table 5). One study found that including more motivated families who self-referred to the trial may result in biased recruitment toward reliable, frequent attendees rather than participants with multiple missed appointments [24]. The importance of caregiver involvement in children and young people intervention was also mentioned because the children and young people–only group did not have better access to digital resources despite the high acceptability and perceived utility of those resources [30]. Another study found that barriers, such as time commitment or accessibility to clinics, influence caregiver decisions regarding children and young people participation [35]. Specifically, children and young people dropped out of the intervention owing to their caregivers’ decisions regarding time and cost, skepticism, and ease of access to the intervention [35]. Furthermore, when the target disease is highly associated with family history (eg, childhood obesity), the attitudes and decision-making of caregivers appear to have an extensive impact on children and young people’s engagement [32].

#### Societal Challenges

Several societal factors (ethnicity, availability of digital technology, sex, clinical settings, and language) were identified as barriers to the generalization of the results (Table 3). Limitations in the recruitment of different ethnicities were described as a challenge in 4 studies, highlighting the need to conduct clinical trials with more diverse ethnic groups [11,17,27,31]. Moreover, the limited availability of digital technology in rural communities or situations requiring social distancing was listed as a challenge in 5 studies [9,15,17,29,40]. In addition, sex differences in internet use patterns and preferences were noted in 2 studies as factors to consider when conducting digital interventions [18,23]. Specifically, boys are more likely than girls to use physical centers rather than web-based centers, requiring higher self-motivation [18,23]. Specific clinical settings (those that enable access to in-person cognitive behavioral therapy) were also considered regarding the generalizability of the outcome or intervention implementation [19,26]. Finally, language barriers were considered a societal challenge in implementing digital interventions, as demonstrated in a study in which non-English speakers were excluded from research [22].

## Discussion

### Overview

Our findings support the growing interest in digital mental health as a field of digital health research. The mental health of children and young people was targeted for digital interventions 3 times more frequently than physical health. In addition, a substantial number of digital interventions directly targeted children and young people. Digital health approaches are expected to provide major benefits to children and young people [44]. However, as children and young people are particularly susceptible to manipulation, targeted digital interventions require a careful risk-benefit analysis based on ethics, regulatory frameworks, monitoring mechanisms, and clinical decision-making.

### Descriptive Characteristics

We found that digital interventions for children and young people are more likely to be delivered via computers than via smartphones. This may be explained by the expansion in smartphone ownership by age; in 2019, more than half of all children and young people aged ≥11 years owned smartphones, whereas less than half of all children and young people aged ≤10 years owned smartphones [45]. On the basis of this expansion, the age of the target user is an essential factor to consider when developing digital interventions.

The duration of digital interventions for children and young people was more likely to vary depending on the target user rather than the target disease. The average duration of digital interventions targeting caregivers was more than twice that of the digital interventions targeting children and young people. Furthermore, the duration of digital interventions targeting children and young people showed the highest variability (from a single session to 24 weeks). The developmental level of children and young people may influence the duration of digital interventions [3]. Therefore, before implementing digital interventions for children and young people, their communication capabilities based on developmental stage must be considered.

In our study, the digital intervention components primarily consisted of guidance and RM. This finding supports the importance of providing children and young people with health education guidance, which can lead to positive well-being, academic success, and healthy adult outcomes [46]. In addition, as engagement in the intervention significantly impacts clinical outcomes [47], RM may be an essential component of digital interventions. Surprisingly, RS was the least used component in children and young people digital interventions. RS may lead to addictive behaviors when misused, similar to smartphones or computer games [48]. Thus, when selecting intervention components for susceptible populations such as children and young people, it is critical to define the component and how it will be applied in the digital intervention.

### Ethical Aspects

Most digital interventions required double consent procedures (children and young people and their caregivers) for real-world implementation. This process is frequently used to protect socially susceptible populations during health interventions. However, under certain circumstances, waiving consent from children and young people or their caregivers may be acceptable, depending on the age and characteristics of the children and young people. In the United States, if children and young people are <7 years, it is possible to waive their consent (as opposed to legal consent) and obtain consent only from their caregivers [49]. In the United Kingdom, children and young people aged <16 years mandate double consent procedures unless they prove maturity based on the Gillick ruling [50]. Although there is no universal consensus on when children should be considered capable of making medical decisions, the possibility that altered decision-making patterns may occur with the onset of adolescence, which occurs at approximately 12 years of age, should be considered [48].

On the basis of our results, consent waivers to caregivers may occasionally be permitted depending on the characteristics of the children and young people. For example, lesbian, gay, bisexual, transgender, queer children and young people are more likely than their peers to encounter stigma in health care settings and rejection from their caregivers [17,51]. This may cause them to be separated and isolated in health care settings, resulting in health disparities. Therefore, logistical barriers may need to be lowered for specific children and young people populations when deploying digital interventions.

On the basis of our findings, 2 types of potential risks are associated with digital interventions for children and young people: adverse events [10,12,14,18,23,28,34,35] and data privacy [10,17,26-28,34,37]. Generally, monitoring and responding to adverse events ensures safety while also advancing our understanding of how users interact with digital interventions. Therefore, it is critical for developers to proactively detect potential safety issues and for regulators to record and process adverse events. For example, the US Food and Drug Administration enforces MedWatch, a web-based reporting system that allows users and health professionals to voluntarily disclose adverse events that occur while using a Food and Drug Administration–regulated product [52]. Currently, the primary risks of digital interventions are privacy, confidentiality, and security of user data and information. Privacy vulnerabilities include unauthorized third-party access to confidential patient-related information, cybercrimes, and accidental data leakage, which could render digital interventions less acceptable to children and young people [31]. To ensure data security and information privacy, digital interventions for children and young people should use a secure platform with deidentified data under different sharable conditions [9-11,14,15,17,22,26-28,34,37].

### Interpersonal Aspects

Regarding the engagement and clinical effectiveness of digital interventions for children and young people, the involvement of their families and caregivers must be considered. The level of engagement of children and young people in digital interventions and clinical trials is frequently influenced by the preferences or barriers of their caregivers [24,30,32,35]. This might be more prevalent when the target disease has a strong family history [32]. This correlates with a recent systematic review that highlighted caregiver involvement as a critical component in treatments and studies of children and young people [53]. Although digital interventions are often likely to increase children and young people’s autonomy and empowerment in their treatment [30], children and young people are susceptible populations that may rely on the decisions of their caregivers, which may differ based on race and ethnicity, sex, or other social determinants of health.

### Societal Aspects

Our findings reveal several societal issues that must be considered when developing and deploying digital interventions for children and young people, including their diversity and accessibility. The diversity of populations and settings in clinical trials is closely linked to the generalizability of outcomes. Although this is a critical point for scalability and implementation of digital interventions, most studies had a limited ethnicity or sex distribution in their recruitment [11,17,18,23,27,31] and settings [19,26]. To overcome these barriers, clinical trials should be conducted iteratively based on the *DTx Real World Evidence Framework* [54]*,* highlighting partnership development as a co-design process and using diverse research methods. Similarly, when implementing interventions, language differences should be considered [22].

Although ease of access is one of the primary benefits of digital interventions, considerable structural barriers exist, such as socioeconomic inequality; this is called the *digital divide*. Many studies report that accessing digital interventions in rural areas is difficult, making it problematic for children and young people to undertake relevant interventions [9,15,17,29,40]. With adequate access, overcoming issues with digital literacy and facing severe disadvantages of novel health care solutions may be easier. To address these issues, policy makers may partner with educational institutions to integrate these technologies into the school setting. School-based health practice has the potential to address unmet children and young people health needs by increasing access to advanced health care for disadvantaged and marginalized populations.

### Limitations

This study had several limitations. First, some studies on digital interventions for children and young people may have been omitted, despite our detailed methodology. However, by combining the keywords used to search the 5 databases, we retrieved additional studies from Google Scholar that were absent from these databases. Second, no quality assessment of the included papers was performed, given the scope of this study. This should be considered in future research to provide an understanding of the quality of current research. Finally, only articles written in English were included in this study.

### Recommendations for Future Research

Researchers may consider involving caregivers in future research on digital intervention for children and young people, which may influence the engagement of children and young people in interventions as well as the prognosis of a disease that affects children and young people. Researchers may also consider setting up an appropriate follow-up safety plan with children and young people and their caregivers after they have left or completed the research with digital interventions as their responsibility to manage safety and deal with any emergencies for children and young people does not end when the research is completed. More research is needed to establish the standard of appropriate secure platform and data-sharing conditions when implementing digital intervention for children and young people by carefully examining and understanding the theory and features of digital interventions that researchers are going to conduct. In addition, future studies may include a clear breakdown of the development and implementation efforts of digital interventions for children and young people because these details are necessary for assessing their effectiveness and accessibility, which will be very useful to other researchers.

### Conclusions

**Figure 3:**
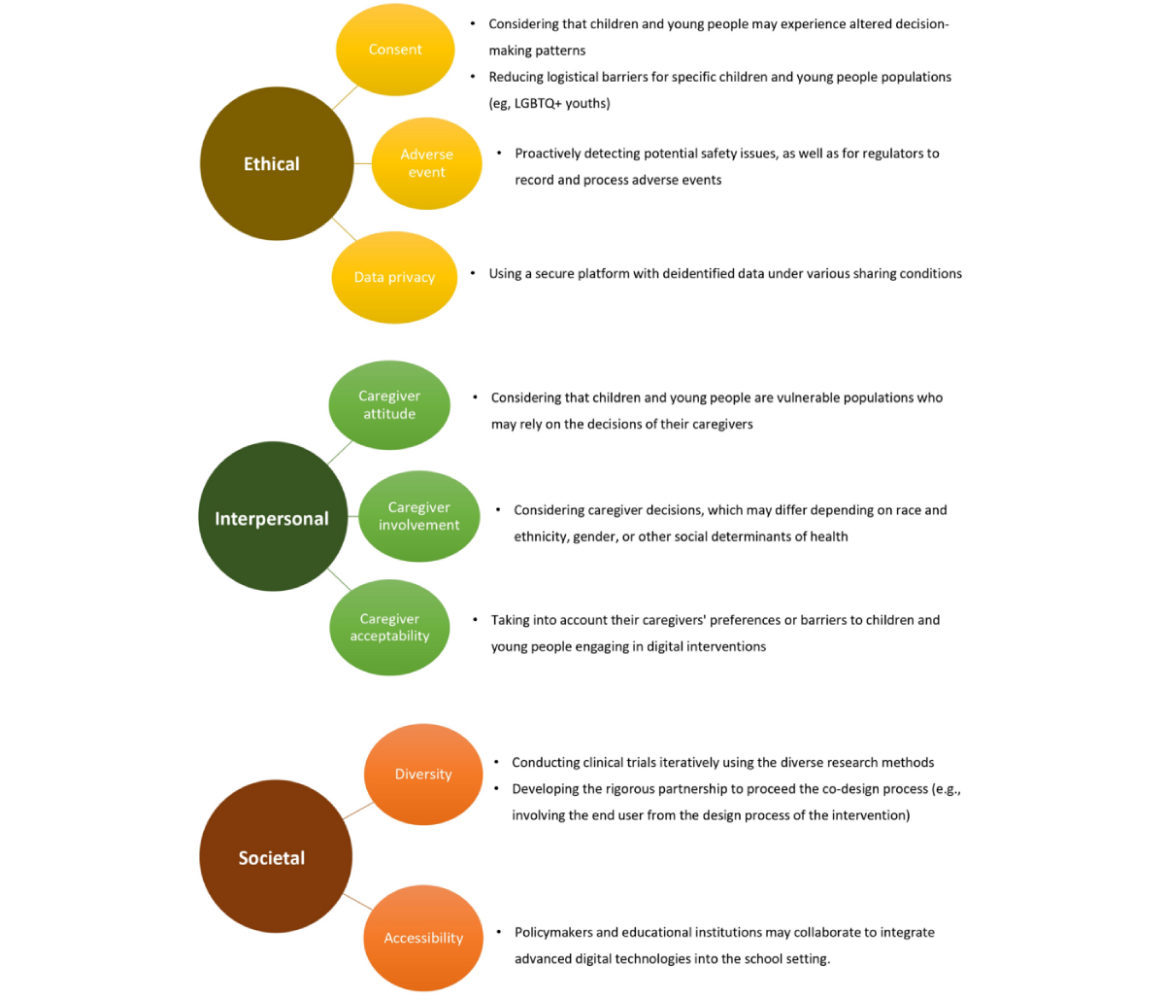
An overview of potential challenges and suggestions for digital-based interventions for children and young people. LGBTQ+: lesbian, gay, bisexual, transgender, queer.

We identified potential challenges based on the distinctive features of children and young people and digital-based interventions published in peer-reviewed journals. We provided suggestions regarding ethical, interpersonal, and societal aspects to consider when developing and deploying such interventions for children and young people (Figure 3). To advance work in this dynamic area, we recommend thoroughly understanding the characteristics of children and young people at every developmental stage and considering their caregivers throughout the intervention period. To produce rigorous real-world evidence, a rigorous partnership for a co-design process that meaningfully involves children and young people and their caregivers in designing and implementing digital health interventions is recommended.

## Appendix

Multimedia Appendix 1 PRISMA-ScR (Preferred Reporting Items for Systematic Reviews and Meta-Analyses extension for Scoping Reviews) checklist.

Multimedia Appendix 2 Search strategy for study selection.

Multimedia Appendix 3 Inclusion and exclusion criteria.

Multimedia Appendix 4 Components of digital interventions for children and young people or caregivers.

## Abbreviations

DTx: digital therapeutics
PRISMA: Preferred Reporting Items for Systematic Reviews and Meta-Analyses
PRISMA-ScR: Preferred Reporting Items for Systematic Reviews and Meta-Analyses extension for Scoping Reviews
RM: reminder and monitoring
RS: reward system
SF: supportive feedback
