# Supplementary material for: Characteristics and Potential Challenges of Digital-Based Interventions for Children and Young People: Scoping Review
Source: J Med Internet Res. 2023 Apr 14;25:e45465. doi: 10.2196/45465 (PMC10148209; doi:10.2196/45465)
Supplement: Multimedia Appendix 2 [file jmir_v25i1e45465_app2.docx]

**Multimedia Appendix 2.** Search strategy for study selection.

| #1 | ("digital*" OR "digital therapeutic" OR "digital intervention" OR "digital technology" OR "digital electronics" OR “digital health” OR “digital sensors” OR "software" OR "mobile*" OR "smartphone" OR "tele*" OR "internet" OR "web" OR "computer") |
| --- | --- |
| #2 | ("mental*" OR "mental health" OR "mental illness" OR “mental disorder” OR “emotional health” OR "cognitive disorder" OR “attention*” OR “cognition*” OR "behavioral disorder" OR "developmental disorder" OR "psychiatric illness" OR "psychiatric disorder" OR “disease” OR “syndrome” OR “medical problems” OR “medical issues”) |
| #3 | ("pediatric*" OR "young*" OR "child*" OR “young adult” OR "adolescent" OR "teenager" OR “caregiver” OR “parent*”) |
| #4 | #1 AND #2 AND #3 |

| **Data source** | **Query (searched on August 19, 2022)** | **Results** |
| --- | --- | --- |
| PubMed | #4 AND ((clinicalstudy[Filter] OR clinicaltrial[Filter] OR randomizedcontrolledtrial[Filter]) AND (fft[Filter]) AND (humans[Filter]) AND (2018/1/1:2022/12/31[pdat]) AND (english[Filter]) AND (allchild[Filter])) | 1097 |
| Scopus | TITLE-ABS-KEY (#4) AND ( LIMIT-TO ( PUBSTAGE, "final" ) ) AND ( LIMIT-TO ( DOCTYPE, "ar" ) ) AND ( LIMIT-TO ( PUBYEAR, 2022 ) OR LIMIT-TO ( PUBYEAR, 2021 ) OR LIMIT-TO ( PUBYEAR, 2020 ) OR LIMIT-TO ( PUBYEAR, 2019 ) OR LIMIT-TO ( PUBYEAR, 2018 ) ) AND ( LIMIT-TO ( EXACTKEYWORD , "Clinical Trial" ) ) AND ( LIMIT-TO ( OA, "all" ) ) AND ( LIMIT-TO ( LANGUAGE, "English" ) ) | 1492 |
| Embase | ('digital*' OR 'digital therapeutic' OR 'digital intervention'/exp OR 'digital intervention' OR 'digital technology'/exp OR 'digital technology' OR 'digital electronics' OR 'digital health'/exp OR 'digital health' OR 'digital sensors' OR 'software'/exp OR 'software' OR 'mobile*' OR 'smartphone'/exp OR 'smartphone' OR 'tele*' OR 'internet'/exp OR 'internet' OR 'web'/exp OR 'web' OR 'computer'/exp OR 'computer') AND ('mental*' OR 'mental health'/exp OR 'mental health' OR 'mental illness'/exp OR 'mental illness' OR 'mental disorder'/exp OR 'mental disorder' OR 'emotional health'/exp OR 'emotional health' OR 'cognitive disorder'/exp OR 'cognitive disorder' OR 'attention*' OR 'cognition*' OR 'behavioral disorder'/exp OR 'behavioral disorder' OR 'developmental disorder'/exp OR 'developmental disorder' OR 'psychiatric illness'/exp OR 'psychiatric illness' OR 'psychiatric disorder'/exp OR 'psychiatric disorder' OR 'disease'/exp OR 'disease' OR 'syndrome'/exp OR 'syndrome' OR 'medical problems' OR 'medical issues') AND ('pediatric*' OR 'young*' OR 'child*' OR 'young adult'/exp OR 'young adult' OR 'adolescent'/exp OR 'adolescent' OR 'teenager'/exp OR 'teenager' OR 'caregiver'/exp OR 'caregiver' OR 'parent*') AND ([controlled clinical trial]/lim OR [randomized controlled trial]/lim) AND [2018-2022]/py AND 'clinical trial'/de AND ([adolescent]/lim OR [child]/lim OR [embryo]/lim OR [fetus]/lim OR [infant]/lim OR [newborn]/lim OR [preschool]/lim OR [school]/lim) | 522 |
| Medline | #4 AND Limiters:   1. Scholarly (Peer reviewed) Journals 2. Full text 3. Date of publication: 20180101-20221231 4. English Language 5. Human 6. Age-Related: All Child: 0–18 years 7. Publication Type: Research, Clinical Trial (Phase 1,2,3), Controlled Clinical Trial, Clinical Trial, Randomized Controlled Trial | 458 |
| CINAHL | #4 AND Limiters:   1. Scholarly (Peer reviewed) Journals 2. Full Text 3. Date of Publication: 20180101-20221231 4. English Language 5. Age Groups: All Infant, All Child 6. Research Article 7. Publication Type: Clinical Trial, Randomized Controlled Trial | 206 |
| **Other sources** | | |
| Google Scholar | Keyword used:  “digital*”, “digital therapeutic”, “digital intervention”, “digital technology”, “digital electronics”, “mental*”, “mental health”, “mental illness”, “cognitive*”, “behavioral*”, “developmental*”, “mental disorder”, “psychiatric illness”, “psychiatric disorder”, “pediatric*”, “young*”, “child*”, “adolescent”, “youth”, “teenager” | - |
