# Supplementary material for: Characteristics and Potential Challenges of Digital-Based Interventions for Children and Young People: Scoping Review
Source: J Med Internet Res. 2023 Apr 14;25:e45465. doi: 10.2196/45465 (PMC10148209; doi:10.2196/45465)
Supplement: Multimedia Appendix 3 [file jmir_v25i1e45465_app3.docx]

| **Multimedia Appendix 3.** Inclusion and exclusion criteria. | | |
| --- | --- | --- |
|  | **Inclusion criteria** | **Exclusion criteria** |
| **Search string** | - Articles searched based on the search string in the following databases (PubMed, Scopus, Medline, Embase, CINAHL) and other source (Google Scholar) | - Duplicates |
| **Retrieved article type** | - Original peer-reviewed articles in scientific journals - Clinical Trials - Full text available | - Review Articles - Study protocols - Books or documents - Abstract only available |
| **Population** | - Children under the age of 19 - Parents and families of the children as caregivers - Experiencing at least one target medical problem | - Target group aged 19 and above who is not a caregiver |
| **Intervention type** | - Digital technology interventions targeting medical problems | - Non-digital interventions |
