# Supplementary material for: Characteristics and Potential Challenges of Digital-Based Interventions for Children and Young People: Scoping Review
Source: J Med Internet Res. 2023 Apr 14;25:e45465. doi: 10.2196/45465 (PMC10148209; doi:10.2196/45465)

| **Multimedia Appendix 4.** Components of digital interventions for CYP or caregivers. | | | | | |
| --- | --- | --- | --- | --- | --- |
| **Author (year)** | **Guidance** | **Task and activity** | **Reminder and monitoring** | **Supportive feedback** | **Reward system** |
| Moor et al. [14], 2019 | O^a^ | O | O | O | -^b^ |
| Kollins et al. [34], 2020 | - | O | O | - | O |
| Sosnowski et al. [9], 2022 | - | O | O | - | O |
| Zheng et al. [15], 2021 | O | O | O | - | - |
| Hanrahan et al. [16], 2020 | O | - | O | - | - |
| Chung et al. [31], 2021 | O | - | O | - | - |
| Ko et al. [36], 2020 | - | O | - | - | - |
| Edridge et al. [39], 2020 | - | O | - | - | O |
| Dobias et al. [17], 2021 | O | - | - | - | - |
| Haug et al. [43], 2020 | - | - | O | O | O |
| Topooco et al. [18], 2018 | O | O | O | O | - |
| Topooco et al. [23], 2019 | O | - | - | O | - |
| Jesus et al. [38], 2019 | - | O | - | - | - |
| Aspvall et al. [19], 2021 | O | - | - | O | - |
| Cliffe et al. [20], 2020 | O | - | O | O | - |
| Voss et al. [10], 2019 | - | O | - | O | - |
| Sourander et al. [21], 2022 | O | O | O | O | - |
| Ranney et al. [22], 2018 | O | - | O | - | - |
| Gallen et al. [37], 2021 | - | O | O | - | - |
| Khan et al. [24], 2021 | O | - | - | O | - |
| Kenny et al. [25], 2020 | O | O | O | - | - |
| Lenhard et al. [26], 2020 | O | O | - | O | - |
| Osborn et al. [40], 2020 | - | O | - | - | - |
| Nordh et al. [28], 2021 | O | - | - | - | - |
| Lindqvist et al. [12], 2020 | O | O | O | O | - |
| Wade et al. [30], 2018 | O | - | - | O | - |
| Palermo et al. [11], 2020 | O | O | O | - | O |
| Hardy et al. [35], 2021 | - | O | O | O | O |
| Klee et al. [42], 2018 | - | - | O | O | - |
| Alfonsi et al. [41], 2020 | - | - | O | - | - |
| Lei et al. [32], 2021 | O | - | O | O | - |
| Shedrief et al. [33], 2021 | O | O | O | O | - |
| Knox et al. [27], 2019 | O | O | O | O | - |
| Schmidt et al. [29], 2022 | O | - | O | O | - |

^a^O: included in the intervention; ^b^-: not included in the intervention.

Abbreviations: CYP, children and young people.


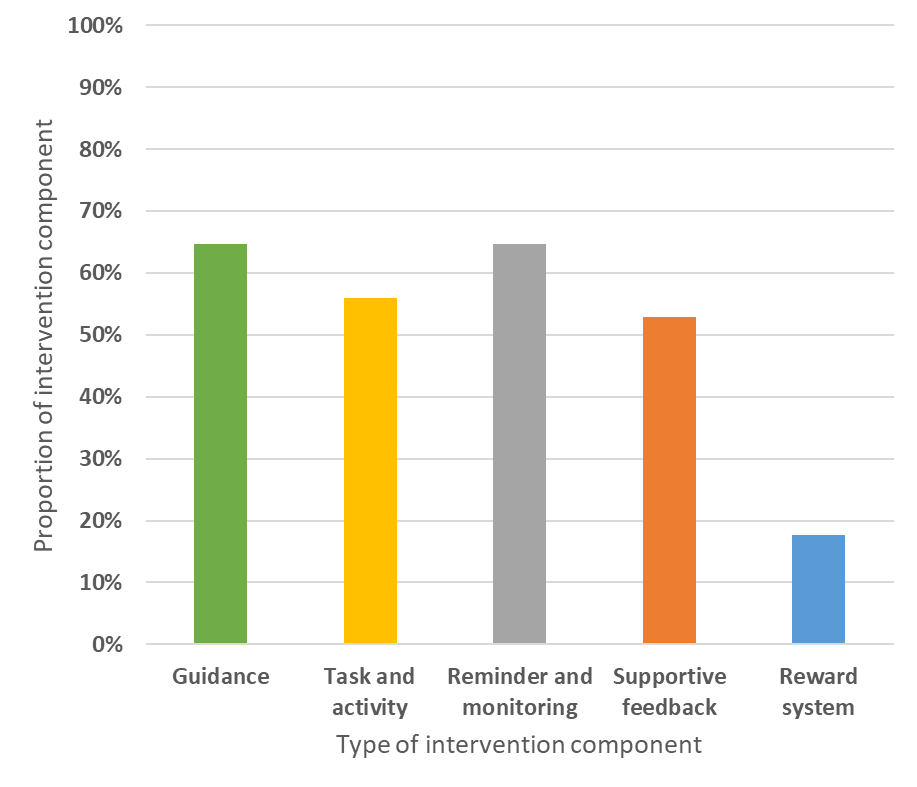

Supplement: Multimedia Appendix 4 [file jmir_v25i1e45465_app4.docx]
